# A call for refining the role of humic-like substances in the oceanic iron cycle

**DOI:** 10.1038/s41598-020-62266-7

**Published:** 2020-04-09

**Authors:** Hannah Whitby, Hélène Planquette, Nicolas Cassar, Eva Bucciarelli, Christopher L. Osburn, David J. Janssen, Jay T. Cullen, Aridane G. González, Christoph Völker, Géraldine Sarthou

**Affiliations:** 1 CNRS, Univ Brest, IRD, Ifremer, LEMAR, F-29280 Plouzané, France; 2 Division of Earth and Ocean Sciences, Nicholas School of the Environment, Duke University, Durham, NC 27708 USA; 3 Marine, Earth, and Atmospheric Sciences, NC State University, Raleigh, NC 27695 USA; 4 Institute of Ocean Sciences, Fisheries and Oceans Canada, 9860 W Saanich Rd, Sidney, BC V8L 5T5 Canada; 5 University of Bern, Institute of Geological Sciences & Oeschger Center for Climate Change Research, Baltzerstrasse 1-3 3012, Bern, Switzerland; 6 School of Earth and Ocean Sciences, University of Victoria, 3800 Finnerty Road, Victoria, BC V8P 5C2 Canada; 7 Instituto de Oceanografía y Cambio Global, IOCAG. Universidad de Las Palmas de Gran Canaria, ULPGC, Parque Científico Tecnológico de Taliarte, 35214 Telde, Spain; 8 Alfred Wegener Institute, Helmholtz Centre for Polar and Marine Research, Am Handelshafen 12, 27570 Bremerhaven, Germany; 9 University of Liverpool, Liverpool, UK

**Keywords:** Marine chemistry, Biogeochemistry, Environmental chemistry

## Abstract

Primary production by phytoplankton represents a major pathway whereby atmospheric CO_2_ is sequestered in the ocean, but this requires iron, which is in scarce supply. As over 99% of iron is complexed to organic ligands, which increase iron solubility and microbial availability, understanding the processes governing ligand dynamics is of fundamental importance. Ligands within humic-like substances have long been considered important for iron complexation, but their role has never been explained in an oceanographically consistent manner. Here we show iron co-varying with electroactive humic substances at multiple open ocean sites, with the ratio of iron to humics increasing with depth. Our results agree with humic ligands composing a large fraction of the iron-binding ligand pool throughout the water column. We demonstrate how maximum dissolved iron concentrations could be limited by the concentration and binding capacity of humic ligands, and provide a summary of the key processes that could influence these parameters. If this relationship is globally representative, humics could impose a concentration threshold that buffers the deep ocean iron inventory. This study highlights the dearth of humic data, and the immediate need to measure electroactive humics, dissolved iron and iron-binding ligands simultaneously from surface to depth, across different ocean basins.

## Introduction

Iron is a key micronutrient to marine microorganisms. Low concentrations of dissolved iron limit primary production in up to 40% of the ocean^1^. Over long timescales, the deep ocean iron inventory is important for global ocean primary productivity^2^, as it can fuel dissolved iron supplied to the surface through winter mixing and diapycnal diffusion. Since ligand complexation is crucial for maintaining iron in solution^3^, fully quantifying the concentration and characteristics of the ligand pool is an essential task. For example, varying the concentrations of iron binding ligands in biogeochemical models has a significant impact on atmospheric CO_2_ calculations^4^. However, the processes controlling the distribution and iron binding capacity of this pool are difficult to constrain. In order to identify the processes regulating iron complexation, it is necessary to consider the controls on key ligand groups, as well as on the wider dissolved organic carbon (DOC) pool to which they contribute. Although many ligand types exist, they are typically separated into classes defined by the strength of their complexes with iron, measured by competition against artificial ligands of known binding constants^5^. This gives an average concentration and binding strength of one or more ligand classes, but not their identity, which must be inferred by comparison to known compounds. Siderophores represent the strongest natural ligands, released by specialised microorganisms to acquire iron^6^. Although a siderophore-like ligand class is often detected^5^, as of yet, direct measurements have only found siderophores to complex less than 10% of iron (though their contribution could be higher)^7,8^. Weaker ligands also play a predominant role in iron cycling and biological uptake^9^. Around 30% of the marine DOC pool is composed of labile polysaccharides^10^, some of which can form weak complexes with iron^11^. The further decomposition of cell-derived products contributes to a longer-lived DOC pool known as humic-like substances, which compose around 50% of DOC^10^ and of which a smaller fraction (around 5% of DOC) can bind to iron^12^.

When only a single ligand class is detected, it is usually of intermediate binding strength throughout the water column^5^. Humic ligands form iron complexes of intermediate strength^13,14^, bracketed by stability constants of the stronger siderophores and weaker polysaccharides. All major sources of iron to the water column also supply humic-like material^15–18^, with terrestrial-derived humics often dominating iron complexation in estuarine and coastal waters^13,14^. Furthermore, the stability of iron-humic complexes allows iron to be transported long distances by ocean currents^19^. However, despite many indications that humic ligands play a key role in the iron cycle, it has thus far proven difficult to explain the relationship between humic substances, iron and the iron ligand pool in ocean waters. This is because humics are themselves a heterogeneous mixture of soluble and colloidal-sized compounds of various origins^14^ and iron-binding behaviour^20,21^. Here, we demonstrate an intrinsic relationship between dissolved iron and humic ligands throughout the water column across multiple open ocean sites. We attempt to further refine the hypothesis for the proposed geochemical control of iron in seawater^13^ by summarising the key processes that may influence this relationship, which have not previously been considered as controls on oceanic dissolved iron distributions.

## Results and Discussion

### Variability in iron binding by marine humics

**Figure 1:**
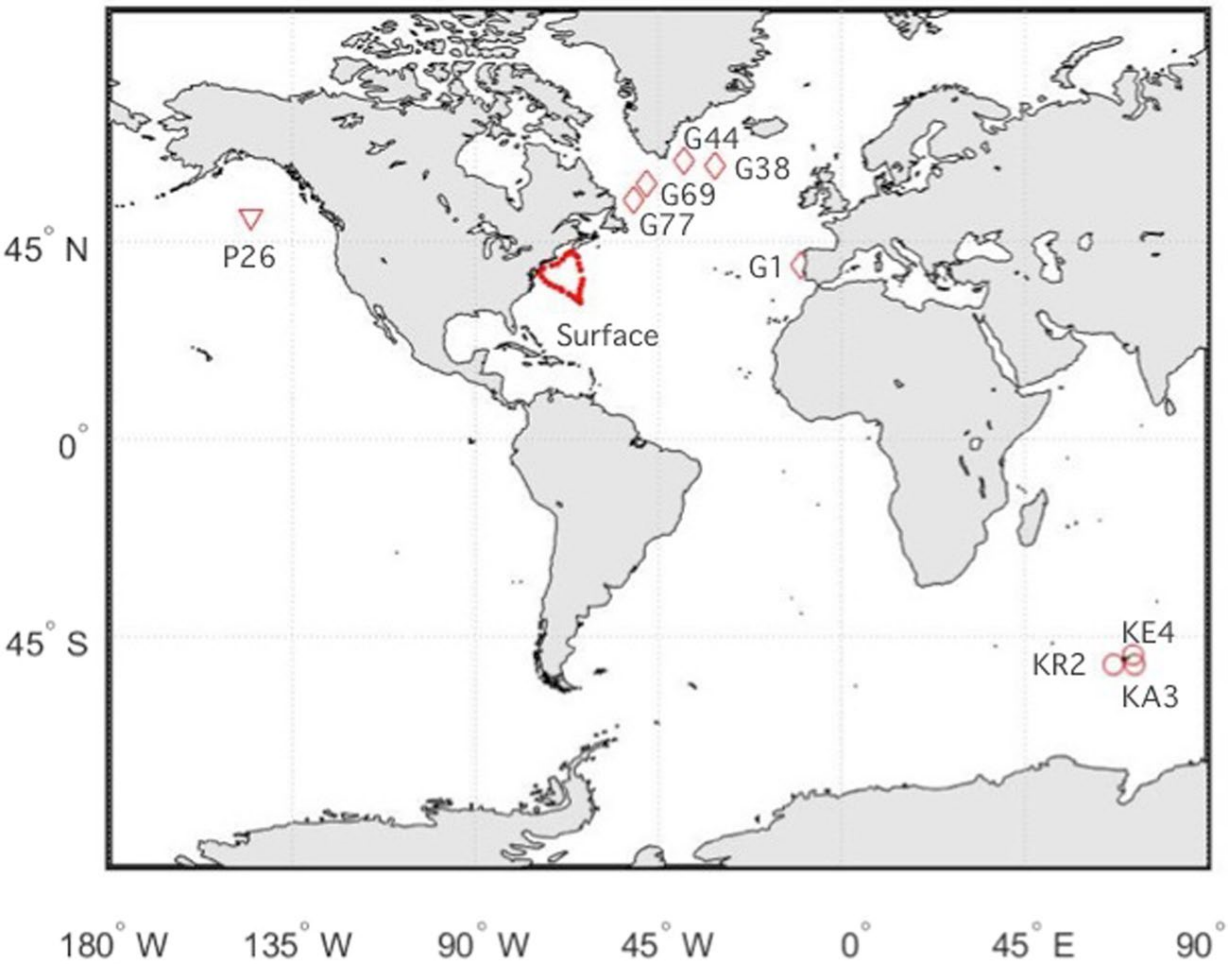
Map of sample locations. Surface samples (5 m depth) are from the Bermuda AE1714 cruise sampled from the Towfish whilst steaming. Depth profiles: Stations G1, G13, G38, G44, G69 and G77 in the North Atlantic are from the GEOVIDE cruise (GA01); P26 in the Northeast Pacific is from the August 2012–13 Line P cruise; KR2, KE4 and KA3 are from the KEOPS2 study in the Southern Ocean (GIpr01). Figure generated using Matlab software.

Sampling locations include the North and Northwest Atlantic, the Southern Ocean and the Northeast Pacific (Fig. 1). Sites range from iron-limited, high nutrient-low chlorophyll regions of the open ocean to iron-replete areas influenced by sea ice and terrestrial inputs. Dissolved metal-binding humic substances were measured electrochemically (eHS) and compared to dissolved iron concentrations. These measurements are distinct from fluorescence-based measurements, and assume that the electroactive humic fraction is representative of the bulk of humic ligands for metals. The highest electroactive humic concentrations were found nearest the coast, in the Northwest Atlantic (reaching 189 μg/L). In open ocean samples, we found similar concentration ranges in all ocean basins (Atlantic 12–116 μg/L, Pacific 18–54 μg/L, Southern Ocean 18–81 μg/L, Supplementary Table S1), with the highest concentrations at intermediate depths. In the North Atlantic, iron-binding ligands and fluorescent dissolved organic matter (FDOM) were also measured.

**Figure 2:**
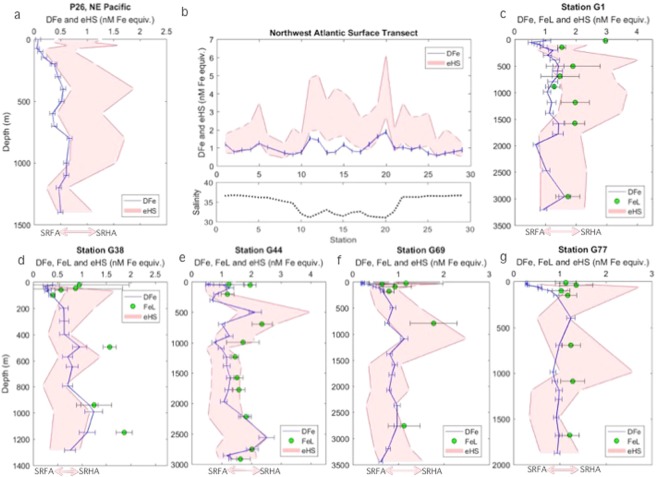
The concentrations of dissolved iron (blue line) and iron-binding ligands (green circles, where available), with an envelope (red) for electroactive humic substances (eHS), encompassing the maximum and minimum iron-binding capacities reported for terrestrial IHSS standards^13,27^. (**a**) Station P26 in the Northeast Pacific (Line P); (**b**) Surface samples (~5 m) Northwest Atlantic (Bermuda AE1714), with salinity included below; (**c–g**) Stations G1-G77 depth profiles from the North Atlantic (GEOVIDE GA01). Error bars show standard deviation, which for eHS is included within the envelope. Spaces in the eHS boundaries show eHS sampling points. Figure generated using Matlab software.

Humic substances are inherently heterogeneous. Composed of a combination of both humic and fulvic acids, which are defined operationally based on extraction protocols, the number and strength of iron binding sites varies^20,21^. Increased aromaticity in humics has been linked to increased iron binding^22^. Terrestrial humics are largely derived from highly aromatic lignins and tannins only found in vascular plants^23^. In contrast, marine humics are mostly derived from plankton and are carboxyl rich and aliphatic, with lower aromatic content^24,25^. While the scarcity of lignin in aquatic systems may account for some discrepancies between terrestrial and marine humics, microbially produced compounds (precursors to humics, such as amino acids, lipids and polysaccharides) from bacteria and algae in both systems may account for some similarities^26^. Since the iron binding capacity of humics is based on numerous factors, here we account for the full range of published iron binding capacities for terrestrial standards from the Suwannee River, using an envelope that encompasses the maximum (Suwannee River Humic Acid, SRHA) and minimum (Suwannee River Fulvic Acid, SRFA) reported values^13,21,27^ as a first approximation (Fig. 2). While marine humics could well incorporate a wider range, the mean iron-binding capacity of our samples (Supplementary Table S1), as well as in the Pacific Ocean^13^ and Mediterranean Sea^12^, indicate a similar range to terrestrial standards, lending confidence to this assumption.

**Figure 3:**
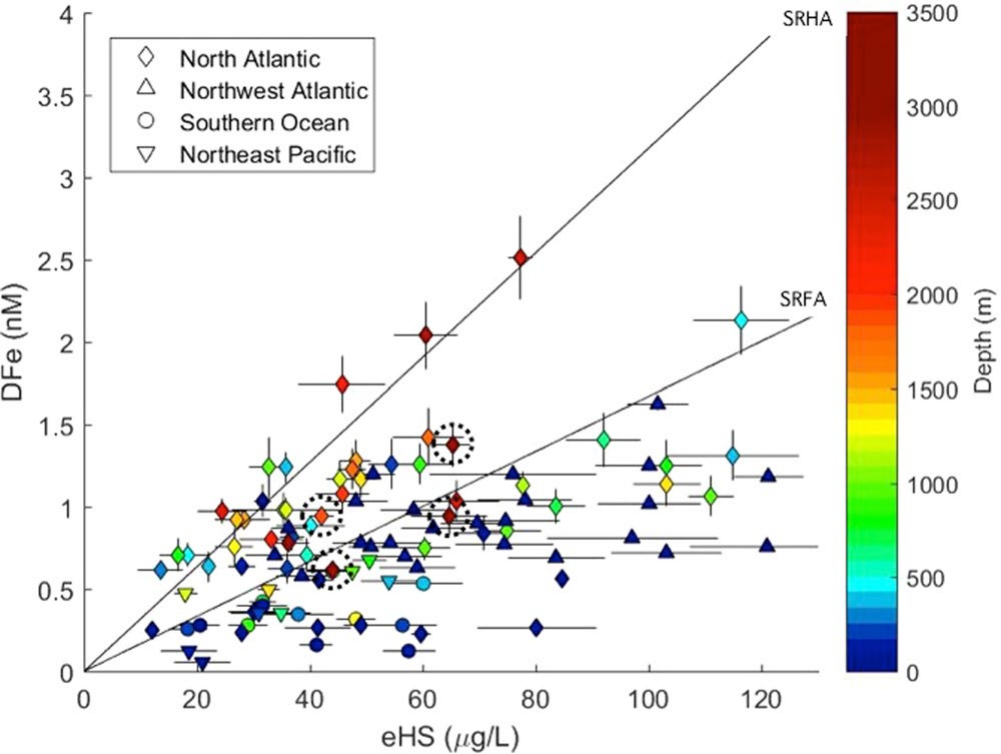
The relationship between the concentrations of electroactive humic substances and dissolved iron from this study, coloured by depth (ρ = 0.5, p < 0.0001, n = 105, where ρ is the Spearman’s correlation coefficient). The upper line demonstrates the maximum iron binding capacity for the measured humic concentrations (32 ± 2.2 nM Fe/mg SRHA), and the lower line shows the lower binding capacity for the equivalent concentration (14.6 ± 0.7 nM Fe/mg SRFA), from reported values for terrestrial IHSS standards^13,27^. These have slopes of 0.33 and 0.73 ± 0.43 as SRHA and SRFA iron-binding equivalents respectively, not shown. The four points with a dashed circle are influenced by sediment, based on transmissiometry data. Error bars represent the standard deviation. Figure generated using Matlab software.

We find broad correspondence between dissolved iron and humic ligands throughout the water column (Fig. 3, ρ = 0.5, p < 0.0001, n = 105, where ρ is the Spearman’s correlation coefficient). However, the range of iron-binding ligands that exist in marine waters, such as low concentrations of siderophores saturated with iron^7^, and the variable iron-binding capacity of humic material, hinder straightforward comparisons. To observe if any broad behavioural trends could be detected and explained, we compared the ratio of dissolved iron to humics (DFe/eHS) across all samples. In surface samples where the organic matter pool is highly dynamic, encompassing coastal to open ocean regions, DFe/eHS covered the full range of iron binding capacities reported for terrestrial standards^13,27^. In 49% of samples shallower than 200 m, DFe/eHS values near or below the lower binding capacity suggested undersaturation of humics with iron. There is a loss of dissolved iron due to scavenging and biological uptake in upper waters. In addition, the humic pool in upper waters is largely aliphatic^28^, and thus has reduced iron-binding capacities. While freshwater inputs can supply aromatic terrestrial humics, photodegradation destroys terrestrial-derived ligands but is a source of aliphatic humics^29^. Humic material in aerosols^17^ and marine-derived organic matter^25^ are also predominantly aliphatic. In a study focussed in the upper 100–200 m in Arctic waters, electroactive humics were found to be on average 62% saturated with iron (range 14–90%), when assuming that all humic ligands had the lowest reported iron binding capacity^27^.

In our samples from the upper 1000 m of the Northeast Pacific and in all samples from the Southern Ocean, where most humic-like material comes from the recent microbial degradation of sinking autochthonous organic matter^30^, the DFe/eHS ratio was below or equivalent to the lowest reported binding capacity of terrestrial humics (Supplementary Fig. S1, Table S1). The humics in these samples thus have low iron binding capacities, or are not saturated with iron; iron concentrations in these samples were generally lower than in our Atlantic samples. Iron saturation of humic ligands has been found to increase with depth^7^, as dissolved iron is supplied to the water column from the bacterial remineralisation of sinking organic matter^3^. In samples deeper than 1000 m in the Pacific, which account for old waters^31^, the DFe/eHS ratio increased respective to surface waters, whereas in the Southern Ocean, DFe/eHS remained low.

In our samples from the North Atlantic, which experienced greater terrestrial influence^32^ and reached deeper sampling depths, DFe/eHS ratios exceeded the lower binding capacity in over 50% of samples. In around 25% of samples, DFe/eHS reached the maximum reported binding capacity of terrestrial humic substances (Fig. 3). Terrestrial humics arrive in the marine environment pre-aged and highly aromatic, particularly those derived from peatlands common in the Arctic. A considerable 30% of lignin phenols discharged by Arctic rivers is exported to the North Atlantic^33^ suggesting some fraction of the humic pool in our North Atlantic samples is likely terrigenous and aromatic^32^. Deep waters reflect an accumulation of aged humic material of higher aromaticity^28,29^, linked to the persistence of terrestrial humics^32,34^, the selective decomposition of organic matter^35^, and the hypothesized aging process of humification^24,28,34^. The increased aromaticity of humics in deep and terrestrially-influenced waters, leading to higher iron binding capacities^22^, could explain the higher DFe/eHS we observe in parts of the North Atlantic.

**Figure 4:**
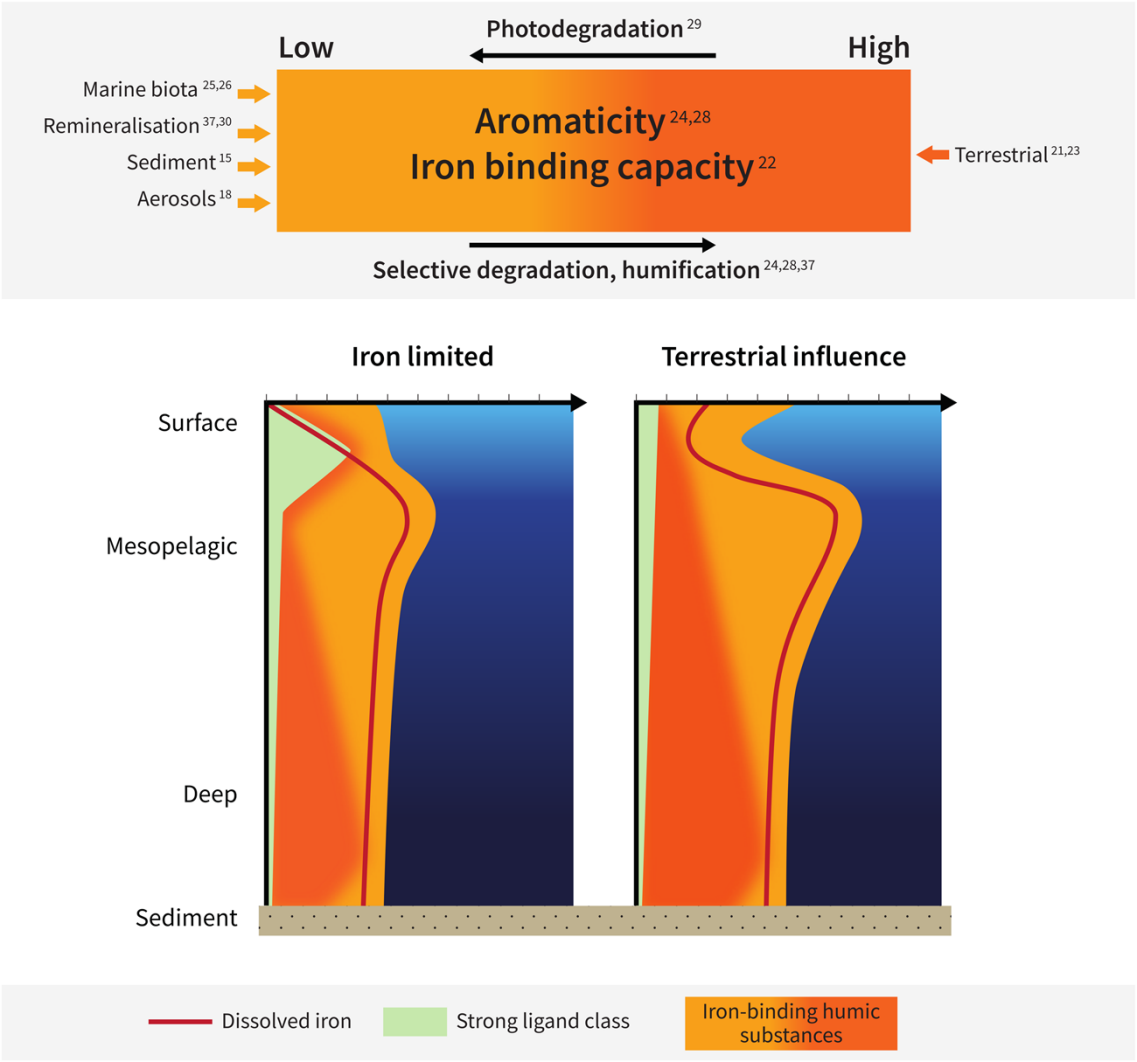
Our proposed schematic of the processes influencing the supply and iron-binding nature of humic substances in seawater. Above represents the continuum of iron binding by humics linked to aromaticity^22^, along with the contributors of humic-like material to ocean waters. Below, our hypothesis for the potential contribution of humics to iron complexation and the iron ligand pool in two scenarios: iron limited regions (left) and terrestrially influenced regions (right). Figure generated using Adobe Illustrator CC software.

Indeed, we find that the binding capacity of humic ligands could define an upper limit for dissolved iron concentrations in the ocean interior (Fig. 3). We find that dissolved iron concentrations do not significantly exceed the maximum potential iron binding capacity of humics in any sample, with many values falling along the upper limit. Our results suggest that a combination of the concentration and binding capacity of humic ligands may control bulk dissolved iron distributions in some parts of the global ocean. Finally, we find lower DFe/eHS near the sediment. Bottom waters are influenced by local microbial communities in sediments supplying fresh aliphatic material^15^, which, along with the increased scavenging of iron onto resuspended particulate material^36^, result in low DFe/eHS. The processes influencing the aromaticity and iron binding capacity of humic ligands are summarised in Fig. 4.

### Metal-binding humic substances: marine and terrestrial influences

As is the case for the wider humic pool, this study finds electroactive humic ligands to be ubiquitous in seawater. Higher concentrations at low salinities demonstrate a terrestrial source, as would be expected. However, elevated concentrations in mesopelagic waters and relatively consistent values at open ocean locations also agree with a marine source and a refractory component. Humic substances are both produced and degraded by bacterial respiration^37^, modifying the optical signature of the humic pool in the thermocline^34^. A proxy for the bacterial remineralisation of organic matter is the apparent oxygen utilisation (AOU). Dissolved iron has been found to correlate with both humic-like fluorescence and the AOU in intermediate and deep waters of the central Pacific^38^. However, it is currently unclear how qualitative fluorescence measurements compare to quantitative electrochemical measurements of humic substances. Only a small fraction of the overall humic pool binds metals, and this may not correspond to the fluorescent fraction. In our samples from the Pacific, dissolved iron showed some agreement with AOU (slope = 0.02 ± 0.14, R^2^ = 0.63, p < 0.01, n = 9, data not shown), though the depth profile highlighted inconsistencies. The DFe/eHS ratio and high AOU in the Pacific was indicative of bacterial remineralisation supplying fulvic-like ligands (Supplementary Fig. S1), however, we found no relationship between electroactive humic concentrations and the AOU.

In the North Atlantic, AOU did not correlate with dissolved iron, electroactive humics, humic-like fluorescence or iron ligands. The North Atlantic has a high terrestrial humic influence, which, along with frequent ventilation, masks the relationship between bacterial remineralisation and organic matter concentrations^30,39^ and thus the relationship with dissolved iron^40^. Our FDOM data agreed with a strong contribution of terrestrial humic-like fluorescence across our North Atlantic samples (Supplementary Table S1). We found no correlation of electroactive humic concentrations with total, terrestrial or marine humic-like fluorescence, suggesting that the fluorescent components of humic material do not always correspond to the metal-binding components. However, metal-binding humics originate from multiple sources, and as the fluorescent properties of marine and terrestrial humics share some common features, their respective contributions are not well distinguished^39^.

Ligand studies in the Atlantic have similarly not found any correlation of the dominant L_1_ and L_2_ ligand classes with AOU^41^, which is also likely in part to be linked to the combination of terrestrial inputs and frequent ventilation^30,39^. Our findings agree with other electrochemical humic and AOU comparisons in the East Atlantic, while a negative correlation was found in the Mediterranean^12^. It was concluded that the negative correlation between electroactive humics and the AOU could be the result of increased degradation of marine-derived humics induced by the enhanced respiration rate of DOC in the Mediterranean, resulting in a humic sink (Dulaquais *et al*. 2018 and references therein). The authors drew attention to the possibility of two pools of humics: one marine-derived pool produced at the surface and available for bacterial degradation, and a second terrestrial-derived pool trapped in the deep sea, which is truly recalcitrant.

Terrestrially-influenced regions experience plumes of elevated dissolved iron concentrations, transported offshore in humic complexes^19^. Peatland sources in particular supply highly aromatic humics. A schematic of our proposed humic contribution to iron binding in iron limited regions compared to waters with a terrestrial signature (Fig. 4) is in agreement with general trends for ligand concentrations and binding strengths^5^. This schematic represents a first approach at highlighting the potential implications of humic aromaticity and variable iron binding capacities on iron complexation in ocean waters. However, it is likely that not all terrestrial inputs are equal, with the iron-binding capacity dependent on the contribution of pre-aged, aromatic components, and the protection of this fraction from degradation during transport. For example, humics from Arctic rivers derived from highly aromatic peatlands and transported during periods of low light intensity may be expected to have higher iron loading than humics from large tropical rivers, where photodegradation and microbial activity reduces the aromaticity of the humic pool during transport. The variability in freshwater iron supply linked to both the concentration and quality of organic matter^22^ deserves further investigation.

When considering that a large fraction of organic matter in the deep ocean is aged and allochthonous^10^, our results suggest that the persistence of terrestrial humics in the deep ocean may be pivotal in the global ocean iron cycle. However, there are many sources of both iron and humic material to seawater and it is likely that aged humics in the deep ocean are derived from a combination of terrestrial^34^, marine^37^ and even hydrothermal sources^16^. Regardless of origin, the apparent upper boundary for dissolved iron concentrations suggests that the concentration and quality of humics could limit bulk dissolved iron concentrations in intermediate and deep waters of the North Atlantic. Our data from the Pacific and Southern Ocean demonstrate a relationship exists between iron and humics in other basins, but a lack of deep samples mean the extent of the upper boundary on iron concentrations remains to be verified in future investigations.

### Humic contribution to the iron ligand pool

**Figure 5:**
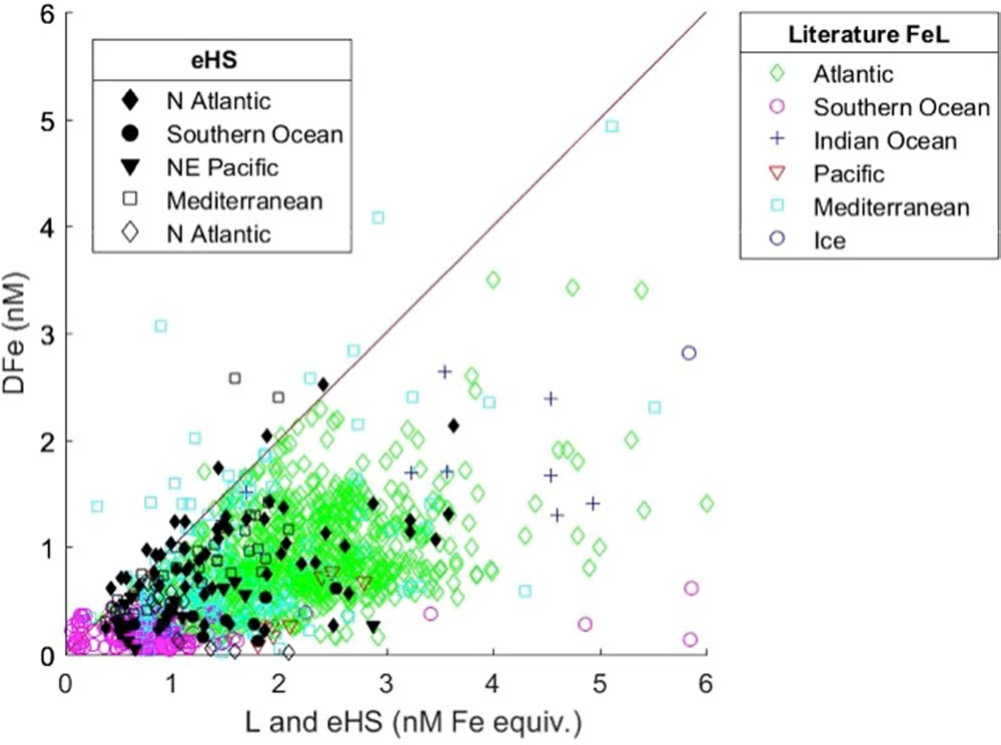
Black shapes show the relationship between the concentrations of dissolved iron (DFe) and humic substances (eHS, from this study, filled, and published values^12^, open) compared to the typical relationship between DFe and the ligand pool (L, open coloured shapes, this study and literature-derived values, slope 0.76 ± 1.3, not shown. See Methods for references). The line represents 1:1 complexation of DFe with the concentration of total ligand or eHS expressed as nanomolar iron binding equivalent, which for eHS is based on the maximum reported potential binding capacity (32 nM Fe per mg/L HA^13^). Figure generated using Matlab software.

As the iron binding capacity of the humic ligands can vary, and as ligand measurements do not distinguish between different compounds of similar binding strengths, constraining the contribution of humics to the iron ligand pool is challenging. In an effort to compare the relationship between dissolved iron and electroactive humics to the typical relationship with the overall ligand pool, we plotted iron ligand data against iron concentrations from an array of published studies, encompassing multiple basins and methods (Fig. 5). Similar plots have been presented previously, including recently in the Pacific^42^, showing a relationship consistent with our figure. It is common to find ligand concentrations in excess of dissolved iron, particularly in surface waters where iron depletion coincides with ligand production. However, the maximum dissolved iron concentrations are almost always limited by the concentration of available ligands, as the poor solubility of iron in seawater results in negligible concentrations of uncomplexed iron^3^.

We compare this established relationship to that of iron and electroactive humics (our study and published values^12^) and find strong similarities. In fact, where dissolved iron exceeds the ligand concentration in the Mediterranean it was acknowledged that the contribution of electroactive humics to the ligand pool may have been underestimated^43^. We also find similar basin-specific features, with concentrations of ligands and electroactive humics in greater excess of iron in the Pacific and Southern Ocean compared to in the Atlantic. We find very good agreement between the slopes of dissolved iron against electroactive humics compared to dissolved iron against published ligand data, 0.73 ± 0.43 and 0.76 ± 0.43 respectively (when using the lower humic iron binding capacity, likely most representative of the bulk of our samples). Similar to previous studies^12,44,45^, we found electroactive humics to correlate with the ligand pool quite well, although samples with data for both parameters were limited. However, all studies find electroactive humic distributions to better agree with dissolved iron directly than with ligand measurements. The ligand pool is dynamic and its composition is unlikely to be constant regionally or with depth. The seawater pH, the molecular size and composition of marine organic matter and the presence of other iron-binding ligands, as well as competition between different metals for common ligands, will all influence the amount of iron complexed by any specific ligand group^44,46^. Furthermore, ligand measurements depend on the detection window and the artificial ligand used, some of which may underestimate humic contributions to the ligand pool^45^ and overestimate the amount of metal complexed by dissolved organic matter^47^, while the effect of buffering the pH during measurements may dampen the influence of subtle pH changes on metal complexation^48^.

Nevertheless, valuable information can be obtained from studying the overall ligand pool. In our North Atlantic samples we find a ligand class of intermediate to strong binding strength throughout the water column (Supplementary Table S1), with the strongest ligands typically at the surface, similar to values reported previously in this region^49^ and in the Southern Ocean^50^. Although we did not have ligand samples for the Pacific, other studies have found the intermediate ligand class dominating iron complexation at depth^42^. In our samples where both parameters were measured, a conservative estimate suggests electroactive humics could account for 23–58% of the ligand pool, to as high as 100% at the highest binding capacity. These values are similar to previous studies in the Mediterranean and Eastern Atlantic, where electroactive humics were found to account for 30–50% of the ligand pool^12^, while they could account for almost all of the ligand pool in the Arctic^45^, with both studies using the lower binding capacity.

In some cases, a siderophore-like class is reported to dominate iron complexation throughout the water column^41^. Recent ligand characterisation techniques in samples off the Californian Coast found the iron ligand pool to largely consist of humic-like material in combination with low concentrations of siderophores, with iron saturation of humics increasing with depth^7^. Thus unsurprisingly, the humic contribution to iron complexation is dependent on the concentration of other, stronger ligand groups, varying regionally and with depth. We find metal-binding humics are ubiquitous and persistent throughout the water column in three ocean basins, corresponding with dissolved iron distributions vertically and laterally. While ligand groups such as siderophores are important for controlling iron bioavailability, humic ligands may control overall dissolved iron distributions, particularly in the deep ocean. The ability of humic ligands to cap dissolved iron concentrations will ultimately be influenced by an array of factors, such as the presence of stronger iron-binding ligands, and the availability of other metals that may compete with iron for humic complexation^44,46^. Even so, we find the maximum dissolved iron concentrations at depth coinciding with the maximum reported binding capacities reported for humic isolates. Processes that control the production, degradation and iron-binding capacity of humic substances may thus be particularly important for controlling dissolved iron distributions in the deep ocean.

### Broader impacts

In light of the central role that marine iron availability may have played in regulating the biological pump during the Pleistocene^51^, a corollary hypothesis is that changes in the delivery of humic ligands and the processes regulating the iron binding capacity (as presented in Fig. 4) may have had an impact on climate. Glacial periods are characterised by increased soil erosion, lower sea level and continental shelf shrinkage, increasing terrestrial organic matter supply to the deep ocean^52^. Glacial-interglacial changes in fluvial and aeolian transport of terrestrial humics to the ocean may thus have played a key role in controlling the marine iron inventory^18,53,54^. However, we know very little about the supply of terrestrial ligands to ocean waters. Currently we do not fully understand the dominant controls on ligands such as humics in the ocean, and whether they persist long enough to influence the iron supply to iron limited regions, or influence its bioavailability to microorganisms. Hence it may be important to better constrain these processes and improve their representation in earth system models if we are to understand and predict climate and climate change.

## Conclusions

Here we present the novel hypothesis that variation in both the concentration and binding capacity of humic material in ocean waters could explain key variations in iron distributions, buffering the dissolved iron inventory of the deep ocean. There is mounting evidence that humics play a significant role in dissolved iron distributions globally. There is therefore a requirement to improve our understanding of this poorly characterised pool in order to constrain the controls on dissolved iron distributions in ocean waters. This study highlights the lack of open ocean data, and an immediate need to simultaneously measure humic, ligand and dissolved iron concentrations throughout the water column in different ocean basins. We recommend studies also consider the effect of physicochemical conditions such as pH on these measurements, and incorporate complementary techniques for measuring and characterising DOC content, in order to better establish the controls on iron complexation in ocean waters.

## Methods

### Sampling

Depth profiles from the North Atlantic were sampled during the GEOVIDE cruise on board the *N/O Pourquoi Pas?* (15 May–30 June 2014, GEOTRACES GA01), in 12 L Teflon-coated GO-FLO (General Oceanics) bottles attached to a 24-bottle powder-coated modified trace metal clean rosette system^55,56^. West Atlantic surface samples were collected using a modified trace metal clean Towfish^57^ pumping seawater directly into a laminar flow hood bubble, during transit of the AE1714 cruise from Bermuda on board the *RV Atlantic Explorer* (July-August 2017). Samples from the Northeast Pacific were collected at station P26 of the Line P Time Series transect from the Canadian Coast Guard Ship *John P. Tully* (14–30 August 2012, cruise 2012–13) into 12 L Teflon-coated GO-FLO (General Oceanics) bottles attached to a 12-bottle powder-coated modified trace metal clean rosette system^55,56^. Samples from the Southern Ocean were collected during the KEOPS2 cruise on board the *N/O Marion Dufresne* (8 October-30 November 2012, GEOTRACES GIpr01) in 10 L externally closing Teflon-lined Niskin-1010X bottles, mounted on a polyurethane powder-coated aluminium frame (TMR, model 1018, General Oceanics)^58^.

Water taken for analysis of dissolved trace metals was either taken from the filtrate of particulate samples (collected on polyethersulfone filters, 0.45 μm Supor) or filtered through a 0.2 μm capsule filter (Sartorius Sartobran 300). Seawater was collected in acid-cleaned 60 mL or 125 mL LDPE bottles after rinsing three times with about 20 mL of seawater. Samples for dissolved iron were acidified to ~pH 1.7 with 2‰ (v/v) HCl (Ultrapur, Merck) in the laminar flow hood. The sample bottles were then double bagged and stored at ambient temperature in the dark before analysis on shore. Samples for ligands and humic substances were double bagged and frozen immediately at −20 °C. Before analysis, the seawater samples were thawed in the dark at 4 °C and measured within 3 days of defrosting. Samples were swirled gently and left to come to room temperature (20 °C) before preparation for analysis.

### Equipment and reagents

Water used for rinsing and dilution of reagents was purified by reverse osmosis (Millipore) and deionisation (Milli-Q, MQ hereafter). Sample bottles (LDPE) were cleaned according to GEOTRACES protocols^59,60^ and stored in MQ for at least a week after acid-cleaning.

All voltammetric measurements used cathodic stripping voltammetry (CSV) in differential pulse mode. The voltammetric apparatus consisted of a µ-Autolab III potentiostat (Ecochemie, Netherlands) connected to a 663 VA stand (Metrohm) with hanging mercury drop electrode (HMDE). The reference electrode was Ag/AgCl with a 3 M KCl salt bridge and a glassy carbon counter electrode. Solutions were stirred with a rotating polytetrafluoroethylene (PTFE) rod. PTFE voltammetric cells used were cleaned using 0.1 M HCl (Suprapur Merck) and rinsed with MQ water. Sample (10 mL) was purged with nitrogen for up to 5 minutes to remove dissolved oxygen prior to analysis.

### Dissolved iron (DFe)

The DFe concentrations from the GEOVIDE (North Atlantic) and Bermuda AE1714 (West Atlantic) were determined by using an online Inductively Coupled Plasma Mass Spectrometry (ICP-MS), at Pôle Spectrométrie Océan (France). The spectrometer was coupled to an ESI SeaFAST pico system to measure dissolved trace metals with a method analytically similar to that of Lagerström *et al*.^61^. Samples collected during KEOPS2 (Southern Ocean) were also analysed by SF-ICP-MS but with a different resin^62,63^. Full details on sampling and measurement are provided by Tonnard *et al*. (2018) and Quéroué *et al*. (2015) respectively. DFe samples from the Pacific were analysed by ICP-MS/MS as described by Jackson *et al*.^64^.

### Humic substances (HS)

We measured the concentrations of electroactive metal-binding HS (eHS) using cathodic stripping voltammetry of their complexes with copper^65^. The copper method was favoured as, unlike iron, dissolved copper can be present in excess of HS. Measurements also do not suffer the same interferences from common marine compounds such as glutathione, as the peak is at a different potential. The concentrations of eHS using the copper technique agree very well with HS concentrations from UV spectrophotometry^65^ and with voltammetric detection of iron-humic complexes directly^44^ in estuarine waters. For this study we also compared the concentrations measured using the copper method^65^ to the recently updated iron method^13,27^ in open ocean waters, surface and deep, and found they agree very well (Supplementary Fig. S2).

Samples from the Atlantic and Southern Ocean were measured with an EPPS buffer (N-(2-hydroxyethyl)piperazine-N′; -2-propanesulfonic acid in 1M NH_4_; 100 μL addition to 10 mL seawater buffered the pH to 8.05) and 30 nM added copper (spectrophotometry standard, pH 2). A deposition potential of +0.05 V was used, usually for a deposition time of 60 s, with a 1s jump to −0.2 V and background subtraction (subtraction of a 1s scan), to improve the baseline and reduce interference from free copper. Samples from the Atlantic were measured using standard additions of the International Humic Substances Society (IHSS) Suwannee River humic acid (SRHA) standard (II 2S101H), and from the Southern Ocean using the IHSS fulvic acid (SRFA) standard (II 2S101F). We performed tests in UV-digested seawater with added known amounts of HS, and found the HA and FA standards to give identical results (as found previously^65^), since the electrochemical methods measure total humic substances and cannot distinguish between HA and FA. Concentrations of eHS for the Pacific samples were already published for copper^66^ and we converted the values to their iron-binding equivalents. The concentration of eHS is measured in mg/L, and the iron-binding equivalent then calculated by multiplication with reported binding capacities. The maximum and minimum Fe-HS binding capacities used for the eHS envelope were derived from published values for IHSS standards: isolated HA bind around 32 nM iron per mg/L^13^, while FA have a reported binding capacity of 14.6–19 nM iron per mg/L^13,21,27^. The lower limit of the envelope was calculated by multiplying the measured eHS concentration by the FA binding capacity, while the upper limit was calculated by multiplication with the HA binding capacity, with upper and lower errors included within the width of the envelope (Eqs. 1 and 2). This envelope provides an assumption of the amount of iron that can be bound by these eHS concentrations as a first approximation based on terrestrial standards, but the range could be wider.

1 $$SRFA=([eHS]\times 14.6)-([eH{S}_{SD}]\times 14.6)$$

2 $$SRHA=([eHS]\times 32)+([eH{S}_{SD}]\times 32)$$

### Iron-binding ligands

We performed iron speciation measurements using CSV with 10 μM 2-(2-Thiazolylazo)-p-cresol (TAC) as the competing ligand^11^. Briefly, 100 μL EPPS buffer was added to 10 mL sample in acid-cleane d PTFE pots (10–12), followed by increasing iron additions (iron spectrophotometry standard, pH 2), usually of 0, 0. 2, 0.4, 0.6, 0.8, 1, 2, 4, 6, 8, 10 nM iron. These were left to stand for one hour before addition of the TAC artificial ligand (final concentration 10 μM), and were then left to equilibrate overnight. The CSV measurement was at a deposition potential of −0.36 V for 180s, followed by a 10s equilibration. At the end of the titration, some samples had fresh additions of 2, 4 and 6 nM iron (final concentrations 12, 14 and 16 nM iron) added to the cell after measurement of the final (+10 nM Fe) pot; this was in order to ensure that the ligands were fully complexed and to check the calculation of the sensitivity, but were not used in the data fitting. Data fitting was performed using the ‘complete fitting’ procedure in independent ProMCC software^67^. These measurements provide a ligand concentration (L) and conditional stability constant (log K_Fe’_) based on the detection window, set by the artificial ligand concentration; this method may miss the contribution of ligands outside of the set detection window, and can underestimate the contribution of humic substances^68^, therefore the values presented represent the minimum concentration of the ligand pool. For the comparison of published concentrations of dissolved iron and iron-binding ligands across multiple ocean basins shown in Fig. 5^11,12,41,45,49,50,69–76^, when more than one ligand class was distinguished, the total ligand concentration was used.

### Optical measurements

Absorbance was measured from 200 to 800 nm on a Varian 300 UV spectrophotometer in 10 cm long quartz cells (Starna Cells, Inc.). MQ water was used as a blank for optical measurements; after blank subtraction, absorbance values (A_λ_) were then converted to Napierian absorption coefficients (a_λ_)^77^:

3 $${{a}}_{{\lambda }}=2.303\frac{{{A}}_{{\lambda }}}{{L}}$$

where *L* is the pathlength, in meters. Fluorescence emission (Em) spectra (300 to 600 nm) were measured at multiple excitation (Ex) wavelengths (240 to 600 nm, in 5 nm increments) on a Varian Eclipse fluorescence spectrometer. Integration time was 0.2 s and excitation and emission slit widths were set to 5 nm. MQ water blanks and quinine sulfate standardization of the detector were conducted each analytical day; results were quantified in Raman units (R. U.). Prior to data analysis, corrections for absorption inner-filter effects and instrument bias were conducted using in-house Matlab code. Emission spectra were concatenated into excitation emission matrices (EEMs) for visualization as contour plots. Values for the various “Coble” peaks (Table 1) were extracted from the EEMs using regional integration over reported wavebands^78^. We used the ratio of different peaks to describe the fulvic and humic nature of HS fluorescence^79^. The marine HS were estimated as the sum of fluorescence under peaks M and N divided by the sum of peaks A, C, M, and N. The terrestrial-like were estimated as the sum of fluorescence of peaks A and C divided by the sum of peaks A, C, M, and N.

**Table 1:** Location in EEM-space of major peaks in DOM fluorescence.

Component	Peak name^80^	Ex/Em	Peak number^81,82^	Source^81,82^	Peak^83,84^
Tyrosine-like, protein-like	B	275/305	8	autochthonous	γ
tryptophan-like, protein-like	T	275/340	7	autochthonous	σ
unknown	N	280/370			
UVC humic-like	A	260/400–460	4	fulvic acid, autochthonous, terrestrial	α′
UVC humic-like	A	260/400–460	1	humic, terrestrial, autochthonous	α′
UVC humic-like	A	260/400–460	3	humic, terrestrial, autochthonous	α′
UVA marine humic-like	M	290–310/370–410	6	anthropogenic wastewater, agriculture	β
UVA humic-like	C	320–360/420–460	5	terrestrial, anthropogenic, agriculture	α
Pigment-like	P	398/660			
UVA humic-like		250(385)/504	2	fulvic acid, terrestrial, autochthonous	

From Coble (2007).

## Supplementary information

Supplementary Information.

Supplementary Table 1

## Data Availability

All data generated or analysed during this study are included in this published article (and its Supplementary Information Files).
