# Supplementary material for: A call for refining the role of humic-like substances in the oceanic iron cycle
Source: Sci Rep. 2020 Apr 9;10:6144. doi: 10.1038/s41598-020-62266-7 (PMC7145848; doi:10.1038/s41598-020-62266-7)
Supplement: Supplementary file 1 — Supplementary Information. [file 41598_2020_62266_MOESM1_ESM.docx]

Supplementary Information for:

**A call for refining the role of humic-like substances in the oceanic iron cycle**

Hannah Whitby*^1^, Hélène Planquette^1^, Nicolas Cassar^1,2^, Eva Bucciarelli^1^, Christopher L. Osburn^3^, David J. Janssen^4,5^, Jay T. Cullen^6^, Aridane G. González^1,7^, Christoph Völker^8^, Géraldine Sarthou^1^

^1^Univ Brest, CNRS, IRD, Ifremer, LEMAR, F-29280 Plouzané, France

^2^Division of Earth and Ocean Sciences, Nicholas School of the Environment, Duke University, Durham, NC 27708, USA

^3^Marine, Earth, and Atmospheric Sciences, NC State University, Raleigh, NC 27695, USA

^4^Institute of Ocean Sciences, Fisheries and Oceans Canada, 9860 W Saanich Rd, Sidney BC, V8L 5T5 Canada

^5^University of Bern, Institute of Geological Sciences & Oeschger Center for Climate Change Research, Baltzerstrasse 1-3 3008, Bern Switzerland

^6^School of Earth and Ocean Sciences, University of Victoria, 3800 Finnerty Road, Victoria BC V8P 5C2

^7^Instituto de Oceanografía y Cambio Global, IOCAG. Universidad de Las Palmas de Gran Canaria, ULPGC, Parque Científico Tecnológico de Taliarte, 35214, Telde Spain

^8^Alfred Wegener Institute, Helmholtz Centre for Polar and Marine Research, Am Handelshafen 12, 27570 Bremerhaven, Germany


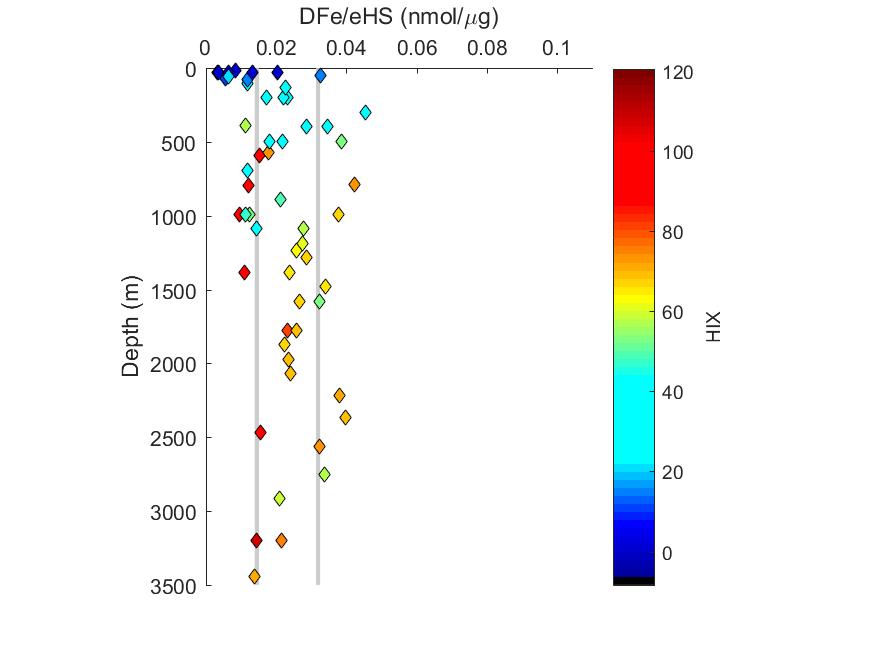

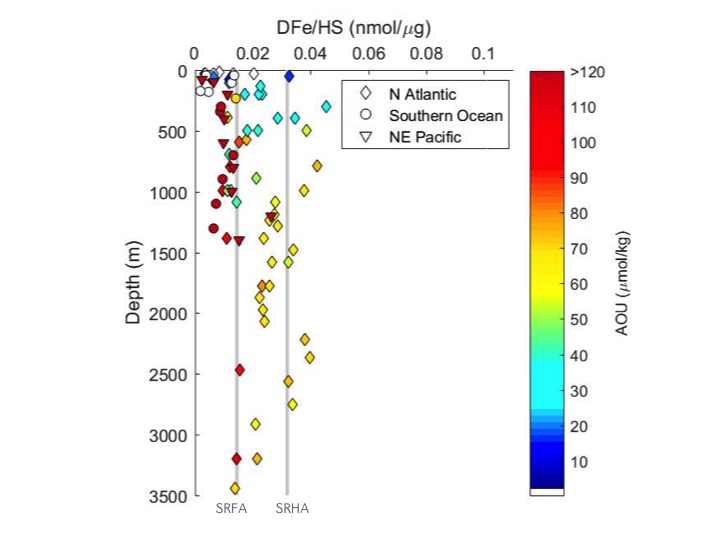


Supplementary Figure 1. The ratio of dissolved iron to electroactive humic substances (DFe/eHS) for all samples, coloured by apparent oxygen utilisation (AOU), a proxy for bacterial remineralisation. AOU coloured from below the maximum mixed layer depth for each region. Lines show the minimum (SRFA) and maximum (SRHA) reported iron binding capacities for terrestrial IHSS standards ([Laglera and van den Berg, 2009](#_ENREF_1); [Sukekava et al., 2018](#_ENREF_2)). Figure generated using Matlab software.

Supplementary Figure 2. A comparison of the concentrations of metal-binding electroactive humic ligands measured using the copper method used in this study([Whitby and van den Berg, 2015](#_ENREF_3)) with values using the updated iron method([Sukekava et al., 2018](#_ENREF_2)), showing good agreement in open ocean waters. From surface (salinity 36.2) and deep ocean waters (depths of 2070, 2365 and 2440 m, all at salinity 34.9). Figure generated using Microsoft Excel software.

Supplementary Table 1 [provided separately as an Excel file]: Concentrations of dissolved iron (DFe), iron ligands (L) expressed in nM Fe equivalent, ligand binding strength (log K_Fe’_) and dissolved electroactive metal-binding humic ligands (eHS) for all stations where measured. DFe/eHS represents an estimate of the iron binding capacity of the humics within the samples (mean = 18.4 ± 11 nM Fe/mg eHS), but assumes saturation of humics with iron and does not consider additional ligands. Chromophoric and fluorescent dissolved organic matter (C/FDOM) was measured in North Atlantic stations only. CDOM abundance (a280) taken as the absorbance coefficient at 280 nm. The marine-derived HS fraction was estimated as the sum of fluorescence under peaks M and N divided by the sum of peaks A, C, M, and N of peaks identified Coble et al. (2007).

**References**

Laglera, L.M. and van den Berg, C.M.G., 2009. Evidence for geochemical control of iron by humic substances in seawater. Limnology and Oceanography, 54(2): 610-619.

Sukekava, C., Downes, J., Slagter, H.A., Gerringa, L.J.A. and Laglera, L.M., 2018. Determination of the contribution of humic substances to iron complexation in seawater by catalytic cathodic stripping voltammetry. Talanta, 189: 359-364.

Whitby, H. and van den Berg, C.M.G., 2015. Evidence for copper-binding humic substances in seawater. Marine Chemistry, 173(0): 282-290.
